# The complete mitochondrial genome of *Hebesoma violentum* (Acanthocephala)

**DOI:** 10.1080/23802359.2018.1473717

**Published:** 2018-05-11

**Authors:** Tingshuang Pan, He Jiang

**Affiliations:** Fisheries Institute, Anhui Academy of Agriculture Sciences, Hefei, China

**Keywords:** Acanthocephala, *Hebesoma violentum*, Rotifera

## Abstract

The acanthocephalan *Hebesoma violentum* Van Cleave was obtained in the intestine of *Siniperca chuatsi*. The complete mt genome sequence of *H. violentum* was obtained by long PCR, containing 36 genes with 12 protein coding genes, 22 transfer RNAs (tRNAs), and two ribosomal RNAs (rRNAs).

Acanthocephalans were recently recognized as a group within the phylum Rotifera in either traditional morphological (Sørensen and Giribet 2006) or molecular phylogenetic analyses (García-Varela and Nadler 2006). In this study, the complete mt genome of *Hebesoma violentum* was sequenced.

*Hebesoma violentum* was dissected out from intestines of *Siniperca chuatsi*, which were captured in Liangzi Lake (30°08′58.1″N, 114°67′36.3″E). The specimen is stored in Fisheries Institute, Anhui Academy of Agriculture Sciences. We amplified mitochondrial DNA using the long PCR method as shown in Pan and Nie (2013). The complete mitochondrial genome of *H. violentum* is 13,393 bp (KC415004).

The genome of *H. violentum* is encoded on the same strand and in the same direction, and they all contain a total of 36 genes and 12 protein-coding genes, including 22 transfer RNAs and two ribosomal RNAs. The 12 protein-coding genes in the mt genome of *H. violetum* also share features in start and stop codons with those in other acanthocephalans.

Phylogenetic analysis was performed using nine of the 12 protein-coding genes by Bayesian inference (BI). The clade containing acanthocephalans and bdelloids is well revealed with high supporting value in the phylogenetic tree. It is indicated that Eoacanthocephala and Palaeacanthocephala are closely related, forming a clade, with Archiacanthocephala as a sister group (Figure 1).

**Figure 1.**
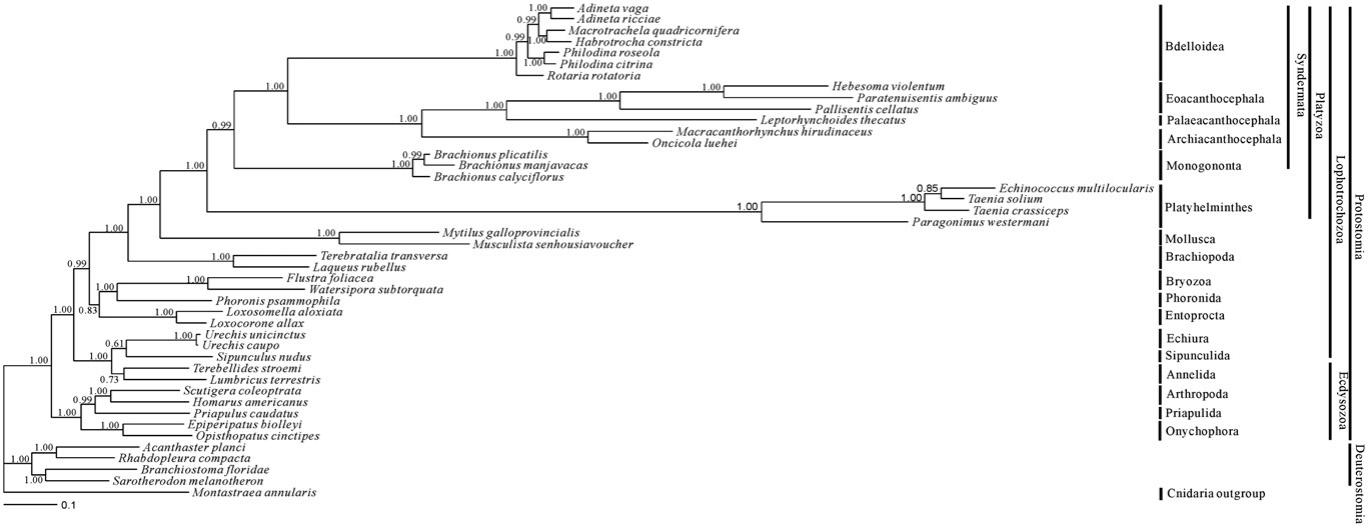
Bayesian phylogenetic tree inferred from amino acid sequence dataset of nine protein-coding genes for 44 metazoan mitochondrial genomes. The tree shows the topology based on concatenated data of nine mitochondrial encoded protein sequences (*cox1*, *atp6*, *nad4L*, *nad4*, *nad5*, *cob*, *nad1*, *cox2*, *cox3*). Reconstruction was performed by MrBayes version 3.2. The numerical values near internal nodes represent Bayesian posterior probability (BPP) values.
